# An Artificial Neural Network Prediction Model for Posttraumatic Epilepsy: Retrospective Cohort Study

**DOI:** 10.2196/25090

**Published:** 2021-08-19

**Authors:** Xueping Wang, Jie Zhong, Ting Lei, Deng Chen, Haijiao Wang, Lina Zhu, Shanshan Chu, Ling Liu

**Affiliations:** 1 Department of Neurology West China Hospital Sichuan University Chengdu China; 2 Department of Ophthalmology Sichuan Provincial People’s Hospital Chengdu China; 3 Department of Neurosurgery Shang Jin Nan Fu Hospital of West China Hospital Sichuan University Chengdu China

**Keywords:** artificial neural network, posttraumatic epilepsy, traumatic brain injury

## Abstract

**Background:**

Posttraumatic epilepsy (PTE) is a common sequela after traumatic brain injury (TBI), and identifying high-risk patients with PTE is necessary for their better treatment. Although artificial neural network (ANN) prediction models have been reported and are superior to traditional models, the ANN prediction model for PTE is lacking.

**Objective:**

We aim to train and validate an ANN model to anticipate the risks of PTE.

**Methods:**

The training cohort was TBI patients registered at West China Hospital. We used a 5-fold cross-validation approach to train and test the ANN model to avoid overfitting; 21 independent variables were used as input neurons in the ANN models, using a back-propagation algorithm to minimize the loss function. Finally, we obtained sensitivity, specificity, and accuracy of each ANN model from the 5 rounds of cross-validation and compared the accuracy with a nomogram prediction model built in our previous work based on the same population. In addition, we evaluated the performance of the model using patients registered at Chengdu Shang Jin Nan Fu Hospital (testing cohort 1) and Sichuan Provincial People’s Hospital (testing cohort 2) between January 1, 2013, and March 1, 2015.

**Results:**

For the training cohort, we enrolled 1301 TBI patients from January 1, 2011, to December 31, 2017. The prevalence of PTE was 12.8% (166/1301, 95% CI 10.9%-14.6%). Of the TBI patients registered in testing cohort 1, PTE prevalence was 10.5% (44/421, 95% CI 7.5%-13.4%). Of the TBI patients registered in testing cohort 2, PTE prevalence was 6.1% (25/413, 95% CI 3.7%-8.4%). The results of the ANN model show that, the area under the receiver operating characteristic curve in the training cohort was 0.907 (95% CI 0.889-0.924), testing cohort 1 was 0.867 (95% CI 0.842-0.893), and testing cohort 2 was 0.859 (95% CI 0.826-0.890). Second, the average accuracy of the training cohort was 0.557 (95% CI 0.510-0.620), with 0.470 (95% CI 0.414-0.526) in testing cohort 1 and 0.344 (95% CI 0.287-0.401) in testing cohort 2. In addition, sensitivity, specificity, positive predictive values and negative predictors in the training cohort (testing cohort 1 and testing cohort 2) were 0.80 (0.83 and 0.80), 0.86 (0.80 and 0.84), 91% (85% and 78%), and 86% (80% and 83%), respectively. When calibrating this ANN model, Brier scored 0.121 in testing cohort 1 and 0.127 in testing cohort 2. Compared with the nomogram model, the ANN prediction model had a higher accuracy (*P*=.01).

**Conclusions:**

This study shows that the ANN model can predict the risk of PTE and is superior to the risk estimated based on traditional statistical methods. However, the calibration of the model is a bit poor, and we need to calibrate it on a large sample size set and further improve the model.

## Introduction

### Background

Traumatic brain injuries (TBIs) reduce patient quality of life and result in high morbidity and mortality [1]. TBI can also lead to a range of sequelae, the most common being posttraumatic epilepsy (PTE), which accounts for 4% to 9% of all epilepsy cases [2-5]. Population-based and cohort studies estimate the overall incidence of PTE ranges from 5% to 50%, especially among war veterans, who receive more penetrating TBIs than civilians [6-12]. Previous literature concludes that the incidence of PTE increases with the severity of TBI [5-7], and the vast majority of PTE appears within the first 2 years after TBI and rises in the following 30 years [3,13].

Because of the high incidence and adverse effects of PTE, clinicians need to identify and better manage those patients at high risk. PTE risk factors such as TBI severity, brain contusion, subdural hematoma, neurosurgery, and early posttraumatic seizure (PTS) are reported by multiple regression methods, etc [14-17]. These results are expressed as risk ratios or odds ratios, but they are inconvenient to use. A lot of TBI patients received antiepileptic prophylaxis to prevent PTE. While clinical trials have shown that antiepileptic prophylaxis within 7 days of TBI reduces the incidence of early seizure attacks, a reduction in PTE has not been seen [18,19]. The negative results of these studies may be due to blind selection of the study population and insufficient follow-up time. In addition, antiepileptic prophylaxis for patients with low risk would add financial burden and side effects, so it is necessary for clinicians to identify those at high risk of PTE. However, so far there are no reliable tools to predict the risk of PTE; if we can predict this via the web, it will be of great significance in the realization of precision medicine [20]. Artificial neural network (ANN) is a form of artificial intelligence that can mimic the problem-solving process of the human brain and generate a mathematical algorithmic model that can handle the nonlinear relationship between variables [21]. ANN is one of the most commonly used methods of supervising machine learning, consisting of 3 layers of neurons: an input layer of independent variables, a hidden layer for processing information, and an output layer for the probability of an outcome. ANN-based risk predictive models have several advantages; they can capture nonlinear relationships among input variables, making them ideal candidates for classifying complex diseases [22,23] such as identifying high-risk transient ischemic attack or minor stroke [24] and assisting in precision medicine for COVID-19 [25]. Compared with logistic regression models, ANN models can predict a complex relationship between variables and are more accurate in classifying the dependent variable [26].

### Aim and Research Questions

To date, no published papers have focused on predicting PTE after TBI through ANN. To investigate this problem, we applied ANN to analyze demographic, clinical, and radiological data from TBI patients to achieve accurate prediction of PTE for individual patients, thereby recognizing PTE patients as early as possible, which might be helpful for further antiepileptogenic intervention studies through identifying the suitable target population. In our previous study, we had built a nomogram model to predict PTE based on the same population, and we wondered whether the ANN model outperformed it.

## Methods

### Design

The study had a retrospective cohort design, and the West China Hospital of Sichuan University Ethics Committee approved this study (no. 2019-936). Subjects or their proxies gave informed verbal consent to participate in this study.

### Participants

This ANN predictive model was developed on a retrospective registry of TBI patients at the West China Hospital (a tertiary referral center in Sichuan province, China) from January 1, 2011, to December 31, 2017. These subjects were the training cohort. The model was also tested in 2 external cohorts registered at Chengdu Shang Jin Nan Fu Hospital (testing cohort 1, n=421) and Sichuan Provincial People’s Hospital (testing cohort 2, n=413) between January 1, 2013, and March 1, 2015. All patients were diagnosed with TBI, which was defined as any hospital discharge with a primary or secondary diagnosis of trauma to the head. According to the *International Statistical Classification of Diseases and Related Health Problems, Tenth Revision* (ICD-10), patient records of those diagnosed with traumatic brain injury (S06.902), cerebral concussion (S06.001), subdural hematoma (S06.501), epidural hematoma (S06.401), traumatic subarachnoid hemorrhage (S06.601), skull fracture (liner or depressed fracture; S02.902), traumatic intracranial hemorrhage (S06.806), brain contusion (S06.201), diffuse axonal injury (S06.204), and open or closed TBI (S06.911) were extracted from the electronic medical record database.

We included all patients with complete demographic, clinical, and radiological data to determine TBI, PTS, and PTE.

**Figure 1:**
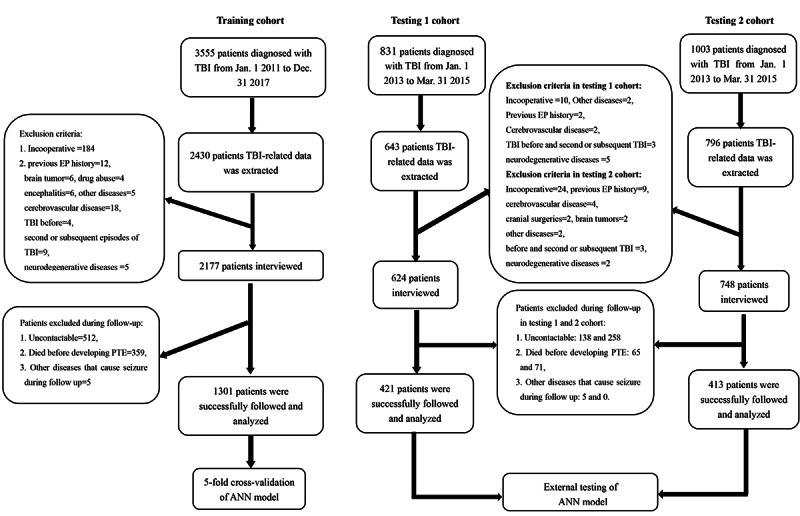
Study cohort. ANN: artificial neural network; TBI: traumatic brain injury

The inclusion criteria were (1) brain injury was caused by an external force, (2) clinical diagnosis of TBI, (3) TBI occurred between January 1, 2011, and December 31, 2017, for West China Hospital patients and for Chengdu Shang Jin Nan Fu Hospital and Sichuan Provincial People’s Hospital patients from January 1, 2013, to March 1, 2015, (4) complete trauma-related data were available in medical records, and (5) patients or their relatives agreed to participate in this study (Figure 1).

The following patients were excluded: (1) patients who had epilepsy or seizures before TBI; (2) patients who had a previous TBI or had second or subsequent episodes of TBI; (3) patients with other conditions that can cause seizure, such as cerebrovascular disease, brain tumors, encephalitis, brain surgery, and other chronic diseases; (4) patients whose general condition was poor or who had other conditions that may lead to epileptic seizures before PTS or PTE came out during follow-up.

### Data and Data Collection

Formally trained neurologists extracted the necessary data for model building from the hospital records of patients. The table included the general condition (age, sex, length of hospital stay, previous history), the clinical and radiological data of TBI (mechanism of TBI, severity of TBI, clinical manifestations, treatments, brain CT performed at initial presentation), and the seizure onset information during their hospitalization (early PTS and immediate PTS). Variables used to construct the predictive model and how we defined and classified them are listed in Table 1.

With the new definition proposed by the International League Against Epilepsy and the International Bureau for Epilepsy, epilepsy requires at least 2 unprovoked (or reflex) seizures occurring more than 24 hours apart and one unprovoked (or reflex) seizure and a probability of further seizures similar to the general recurrence risk (at least 60%) after 2 unprovoked seizures, occurring over the next 10 years [27]. PTS was defined as a single, nonrecurrent convulsive episode that fits in 1 of 3 categories according to the time of seizure onset: immediate PTS, occurring in the first 24 hours following injury; early PTS, occurring more than 24 hours following injury and within 7 days; and late PTS, occurring more than 1 week after trauma. In our study, PTE refers to one or more recurrent seizures occurring more than 1 week after TBI, including late PTS.

**Table 1:** Comparison of demographic data between the posttraumatic epilepsy and non–posttraumatic epilepsy patients in the training cohort.

Variable				Total (n=1301)			Non-PTE^a^ (n=1135)			PTE (n=166)			*P* value
**Demographic data**				—^b^			—			—			—
	Sex, male, n (%)		983 (75.6)			850 (74.6)			133 (82.6)			.03	
	**Age (years), mean (SD)**		38.12 (23.82)			37.79 (24.4)			40.41 (19.09)			.19	
		≤15, n (%)	297 (22.8)		77 (24.3)			20 (12.4)			<.01		
		16-40, n (%)	358 (27.5)		304 (26.7)			54 (33.5)			—		
		41-64, n (%)	447 (34.4)		373 (32.7)			74 (46.0)			—		
		≥65, n (%)	199 (15.3)		186 (16.3)			13 (8.1)			—		
	Follow-up (months), mean (SD)		71.49 (22.54)			72.37 (21.98)			65.6 (25.5)			.08	
**Clinical characteristics**				—			—			—			—
	**GCS^c^** **score, mean (SD)**		11.72 (3.8)			12.22 (3.58)			8.17 (3.40)			.008	
		13-15, n (%)	794 (61)		768 (67.4)			26 (16.1)			<.001		
		9-12, n (%)	227 (17.4)		190 (16.7)			37 (23)			—		
		3-8, n (%)	280 (21.5)		182 (16)			98 (60.9)			—		
	LOH^d^ (days), mean (SD)		14 (23.64)			12.31 (22.17)			25.91 (29.63)			<.001	
	Neurological deficits, n (%)		386 (29.7)			293 (25.7)			93 (57.8)			<.001	
	LOC^e^, n (%)		666 (51.2)			545 (47.8)			121 (75.2)			<.001	
	**LOC time, n (%)**		—			—			—			<.001	
		0-30 minutes	812 (62.4)		782 (68.6)			30 (18.6)			—		
		31 minutes-24 hours	113 (8.7)		96 (8.4)			17 (10.6)			—		
		>24 hours	376 (28.9)		262 (23)			114 (70.8)			—		
	**Etiology of TBI^f^** **, n (%)**		—			—			—			.06	
		MVA^g^	529 (40.7)		445 (39)			84 (52.5)			—		
		Violence	135 (10.4)		121 (10.6)			14 (8.7)			—		
		Fall ≤1 m	376 (28.9)		341 (29.9)			35 (21.7)			—		
		Fall >1 m	261 (20.1)		233 (20.4)			28 (17.4)			—		
	**Treatment, n (%)**		—			—			—			<.001	
		Conservative	651 (50)		602 (52.8)			49 (30.4)			—		
		Neurological surgery	572 (44)		468 (41.1)			104 (64.6)			—		
		Puncture	78 (6)		78 (6)			8 (5)			—		
**Neuroimaging results, n (%)**				—			—			—			—
	SDH^h^		548 (42.1)			455 (39.9)			93 (57.8)			<.001	
	EDH^i^		422 (32.4)			386 (33.9)			36 (22.4)			.004	
	ICH^j^		410 (31.5)			318 (27.9)			92 (57.1)			<.001	
	SAH^k^		336 (25.8)			280 (24.6)			56 (34.8)			.04	
	DAI^l^		95 (7.3)			68 (6)			27 (16.8)			<.001	
	**Contusion site, n (%)**		—			—			—			<.001	
		None	720 (55.3)		679 (59.6)			41 (25.5)			—		
		Frontotemporal lobe	240 (18.4)		160 (14)			80 (49.7)			—		
		Other	341 (26.2)		301 (26.4)			40 (24.8)			—		
	**Fracture, n (%)**		—			—			—			.005	
		No	704 (54)		634 (55.6)			70 (43.5)			—		
		Liner	487 (37.4)		408 (35.8)			79 (49.1)			—		
		Depressed	110 (8.5)		98 (8.6)			12 (7.5)			—		
	Open		79 (6.1)			68 (6)			11 (6.8)			.60	
**PTS^m^** **and PTE, n (%)**				—			—			—			—
	IPTS^n^		13 (1)			11 (1)			2 (1.2)			.87	
	EPTS^o^		74 (5.7)			39 (3.4)			35 (21.7)			<.001	
	**PTE**		166 (12.8)			—			—			—	
		Risk of PTE within 1 year	97 (7.5)		—			—			—		
		Risk of PTE within 5 years	145 (11.1)		—			—			—		
		Risk of PTE within 8 years	166 (12.8)		—			—			—		
		PTE in mild TBI	28 (2.2)		—			—			—		
		PTE in moderate TBI	39 (3.0)		—			—			—		
		PTE in severe TBI	99 (7.6)		—			—			—		

^a^PTE: posttraumatic epilepsy.

^b^Not applicable.

^c^GCS: Glasgow Coma Scale.

^d^LOH: length of hospital stay.

^e^LOC: loss of consciousness.

^f^TBI: traumatic brain injury.

^g^MVA: motor vehicle accident.

^h^SDH: subdural hematoma.

^i^EDH: epidural hematoma.

^j^ICH: intracranial hemorrhage.

^k^SAH: subarachnoid hemorrhage.

^l^DAI: diffuse axonal injury.

^m^PTS: posttraumatic epilepsy.

^n^IPTS: immediate posttraumatic seizure.

^o^EPTS: early posttraumatic seizure.

### Follow-Up and Data Collection

All participants were followed for at least 1 year to monitor for seizures. Among the participants who were unable to understand the survey, we interviewed their close relatives and their general practitioner. The follow-up investigations contain the general condition of the patient (whether there was cachexia or other diseases that can lead to misdiagnosis of PTE), the occurrence of seizures (when did the first seizure attack appear after discharge from the hospital), the type and frequency of seizures (the clinical manifestations and frequency of epileptic seizures), and the treatment condition (whether they took antiepileptic drugs and the drug dosage). If patients or their caregivers reported a seizure attack, neurologists in our team would interview them face-to-face and determined the diagnosis by the definition of PTE according to their clinical manifestations and electroencephalogram results. The main outcome measure was the incidence of PTE.

### Statistical Analysis

#### ANN Model

**Figure 2:**
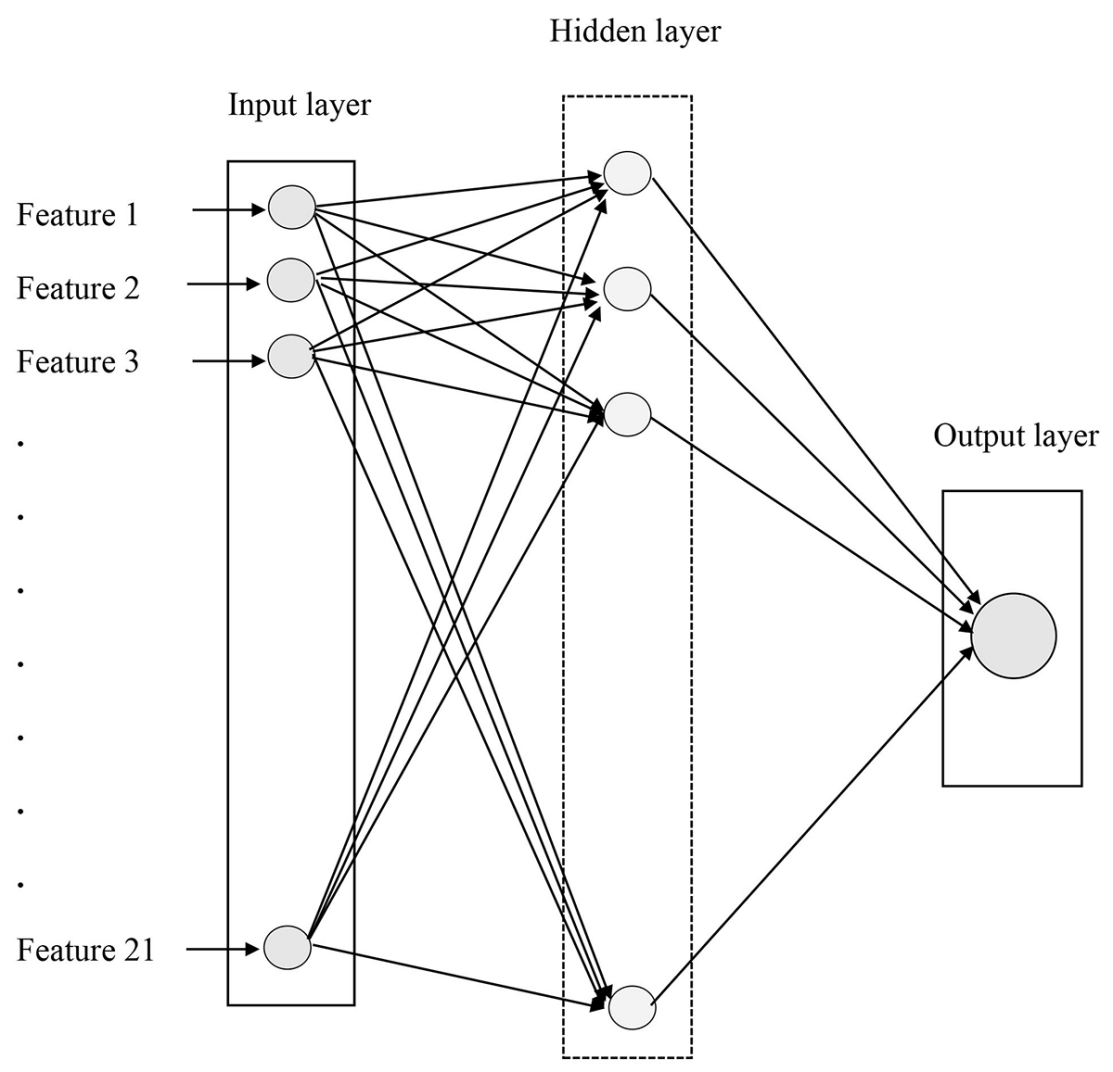
Optimal network architecture of the artificial neural network: a multilayer perceptron.

The most common 3-layer multilayer perceptron ANN model was employed in this study (Figure 2). The input layer incorporated 21 independent variables (Table 1). We performed 5 rounds of model learning and validation (step 1) and calculated the average area under the curve (AUC) using the results of 5 model validations, which represented an estimate of the accuracy of the model. Model test (step 2) was performed in another 2 sets.

##### Step 1: Model Development and Validation

We developed the ANN model using the Keras framework with Python 3.6 programming language (Python Software Foundation). The learning algorithm was back-propagation. Back-propagation can minimize the loss function by iteratively updating the weights between neurons, maximizing the predictive power of the ANN model for the main results. We constructed a cost-sensitive support vector machine classification prediction mode by setting those factors related to PTE as input variables and PTE as an output variable. Given the small sample size, we used a 5-fold cross-validation to validate the ANN models to avoid overfitting [28,29]. The training dataset was randomly divided into 5 folds, and we performed 5 rounds of training and validation of the ANN models. During the 5-fold cross-validation process, 4 folds were the training subsets and the remaining fold was the validation subset, each of which was used only once as a validation set. We first obtained the model index from 5-fold cross-validation, selected the hyperparameters through training, and then retrained the full amount of training data with the optimal parameters of the optimal model. After many attempts, we finally identified 43 (2 × 21 + 1) hidden neurons with 5000 training rounds to train the entire training set. The model began to enter the overfitting phase when the number of training rounds exceeded 5000 epochs.

##### Step 2: Model Test

The predictive performance of the final ANN model was evaluated using 2 external testing datasets (Shang Jin Nan Fu Hospital and Sichuan Provincial People’s Hospital) unknown to the training models. The testing datasets were used for final model evaluation after cross-validation process, model fit, and probability calibration. After setting the ANN hyperparameters, we started to train the neural network with the full amount of training data and stopped training after reaching 5000 times. The test datasets are predicted for about 50 times using a trained neural network, and the results of each prediction were recorded.

#### Evaluating Prediction Accuracy

The performance of the ANN model was measured by its accuracy, sensitivity, specificity, positive predictive value (PPV), and negative predictive value (NPV). Since the primary outcome was a binary variable (PTE or not), area under the receiver operating characteristic curve (ROC), referred to as AUC, was used to assess the accuracy of this predictive model. The average precision, equivalent to the area under the precision-recall curve, was measured for evaluating model performance. The 95% confidence intervals were determined by 50 rounds of model testing (random sampling in the test dataset, learning and verification, and then repeat multiple times). Model calibration was assessed in 2 testing cohorts by calculating Brier scores to examine how well the model predicting PTE frequencies matched the observed one.

Statistical analysis was performed using SPSS (version 22.0, IBM Corp). Independent sample *t* tests were used to compare quantitative data with a normal distribution; otherwise the Mann-Whitney *U* test was applied. The results were presented as mean and standard deviation or interquartile range. The incidence rates were expressed in percentile; to examine associations of categorical and quantitative prognostic factors with the development of PTE, the Fisher exact test and Mann-Whitney *U* test were applied, respectively. In our previous work, by using the rms package in R (version 3.5.1, R Foundation for Statistical Computing), a nomogram was formulated with 7 independent risk factors of PTE founded with multivariate Cox proportional hazards regression analysis based on the cohort of West China Hospital. We compared the prediction accuracy of ANN model with nomogram model [30] using a DeLong test. *P* values reported are 2-tailed, and a value *P*<.05 was considered significant.

## Results

### Patient Characteristics

A total of 2135 patients were included in this study. Between January 1, 2011, and December 31, 2017, 1301 subjects from West China Hospital were enrolled as the training cohort, and the prevalence of PTE was 12.8% (166/1301, 95% CI 10.9%-14.6%). From January 1, 2013, to March 1, 2015, 421 patients from Shang Jin Nan fu Hospital were testing cohort 1, and the prevalence of PTE was 10.5% (44/421, 95% CI 7.5%-13.4%). A total of 413 patients from Sichuan Provincial People’s Hospital were testing cohort 2, and the prevalence of PTE was 6.1% (25/413, 95% CI 3.7%-8.4%). The prevalence of PTE among 3 cohorts had significant difference (*P*=.001).

The comparison of demographic data, clinical manifestation, and radiological results between the PTE- and non-PTE groups in the training cohort was listed in Table 1. Significant differences were found in many variables, including sex, age group, length of hospital days, etiology of TBI, loss of consciousness time, treatment, subdural hematoma, intracranial hemorrhage, diffuse axonal injury, contusion load, contusion site, fracture, and early PTS (both *P*<.001 for all variables). There was no significant difference in follow-up time, subarachnoid hemorrhage, epidural hematoma, open TBI, and intermediate PTS between patients with PTE and non-PTE.

### ANN Predictive Model Performance

**Figure 3:**
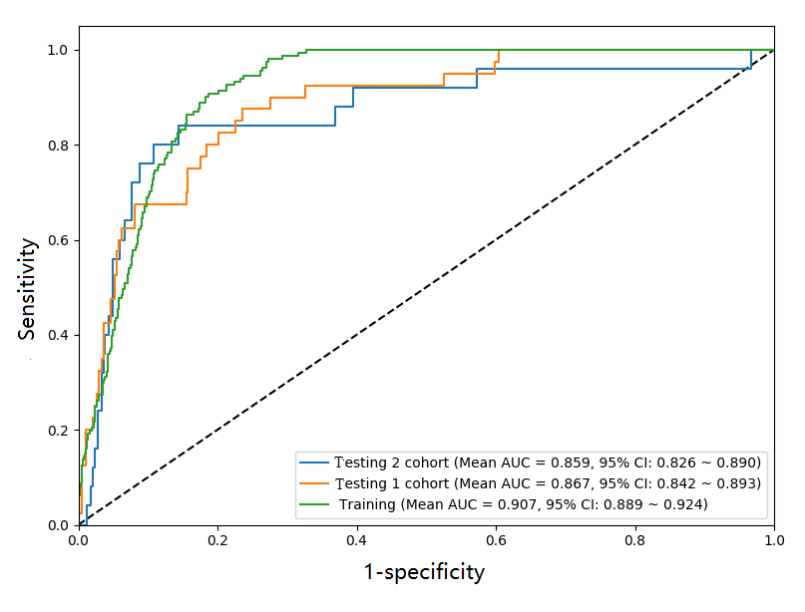
Evaluation of artificial neural network prediction model accuracy using receiver operating characteristic. ANN: artificial neural network; AUC: area under curve.

The ANN prediction model incorporated 21 features from each patient in the training cohort to predict whether an individual would develop PTE. In the training cohort, the mean AUC of the ANN model was 0.907 (95% CI 0.889-0.924), the sensitivity and specificity were 0.80 and 0.86, the PPV and NPV were 91% and 86%, which means 91% of patients who developed PTE and 86% of patients who did not develop PTE were exactly predicted by the ANN model. When testing the ANN model with datasets from Shang Jin Nan Fu Hospital and Sichuan Provincial People’s Hospital, the AUCs were 0.867 (95% CI 0.842-0.893) and 0.859 (95% CI 0.826-0.890), sensitivity was 0.83 and 0.80, specificity was 0.80 and 0.84, PPV was 85% and 78%, and NPV was 80% and 83%. The greater the value of the AUC, the better the performance of the model, which means higher predictive accuracy in this study. Figure 3 shows the ROC of the training cohort and 2 testing cohorts.

**Figure 4:**
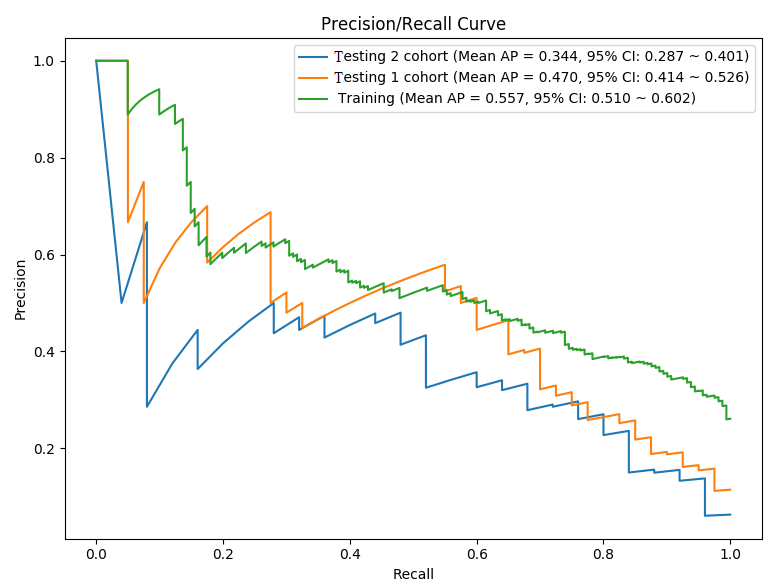
Evaluation of the artificial neural network prediction model accuracy using precision-recall curves. ANN: artificial neural network; AP: average precision.

In addition, the average precision of the ANN prediction model in training cohort was 0.557 (95% CI 0.510-0.620), while the average precision was 0.470 (95% CI 0.414-0.526) in testing cohort 1 and 0.344 (95% CI 0.287-0.401) in testing cohort 2 (Figure 4). Similar to AUC, the larger the average precision value, the better the prediction accuracy. However, unlike the ROC, the area under the precision-recall curve was less than 0.5, which did not mean that the prediction performance of the model was poor. An asymmetric data distribution (ie, the number of negative, or non-PTE, events is much more than the number of positive, or PTE, events) leads to a low overall decrease in average precision and will have a great impact on the precision-recall curve but no effect on the AUC curve [31].

**Figure 5:**
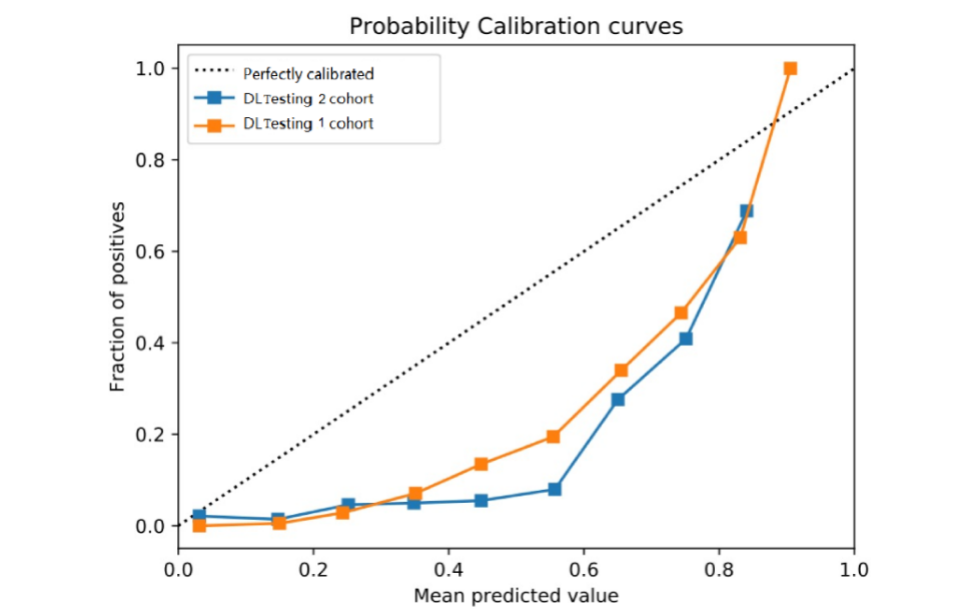
Risk calibration curves for artificial neural network prediction model in two testing cohorts. A curve closer to the dotted diagonal line indicates better calibration, with corresponding lower Brier score. ANN: artificial neural network.

Brier scores were calculated to evaluate the calibration of the ANN prediction model. With the Brier score, we can know the calibration of the prediction model [32]. It ranges from 0 to 1; the lower the Brier score, the better the calibration, so the ideal Brier score is 0, indicating the prediction is completely accurate [33]. When testing our ANN prediction model, the Brier scores were 0.121 in testing cohort 1 and 0.127 in testing cohort 2. Figure 5 shows the calibration plots that compare the proportion of PTE patients predicted by the ANN model with the actual observed rate of PTE. The diagonal curve represents a perfectly calibrated prediction, and the calibration curve should be as close to this diagonal curve as possible. In our study, the calibration curves in the 2 testing groups were a little far away from the diagonal curve, and we needed to calibrate this ANN prediction model with a large-sample dataset. Table 2 showed the performances of the ANN model on the training and 2 testing sets.

**Table 2:** Performances of artificial neural network on training and two testing sets.

Indicator	Training cohort	Testing cohort 1	Testing cohort 2
AUC^a^ (95% CI)	0.907 (0.889-0.924)	0.867 (0.842-0.893)	0.859 (0.826-0.890)
Sensitivity	0.80	0.83	0.80
Specificity	0.86	0.80	0.84
AP^b^ (95% CI)	0.557 (0.510-0.620)	0.470 (0.414-0.526)	0.344 (0.287-0.401)
PPV^c^, %	91	85	78
NPV^d^, %	86	80	83
Brier score	—^e^	0.121	0.127

^a^AUC: area under the curve.

^b^AP: average precision.

^c^PPV: positive predictive value.

^d^NPV: negative predictive value.

^e^Not applicable.

### Compared With Nomogram Prediction Model

In our previous work, we built a nomogram model to predict the risk of PTE with the same training cohort through R statistical analysis [30]. The AUC of this nomogram prediction model was 0.859 (95% CI 0.826-0.891) and sensitivity and specificity were 0.867 and 0.738, respectively. Compared with the traditional nomogram prediction model, the AUC value of the ANN prediction model was higher (0.907, 95% CI 0.889-0.924; *P*=.01), which indicated the ANN model had a higher prediction accuracy.

## Discussion

### Principal Findings

ANN is applied to develop a risk prediction model and is superior to traditional prediction models. In this study, we developed a PTE predictive model using ANN methods, which involved 21 predictors (listed in Table 1). In the training cohort, the ANN model had an accuracy of 0.907, average precision of 0.557, sensitivity of 0.80, specificity of 0.86, PPV of 91%, and NPV of 86%. For testing cohort 1 (testing cohort 2), this model had an accuracy of 0.867 (0.859), average precision of 0.470 (0.344), sensitivity of 0.83 (0.80), specificity of 0.80 (0.84), PPV of 85% (78%), NPV of 80% (83%), and Brier score 0.121 (0.127), suggesting that this ANN model was valuable. To our knowledge, this is also the first study to develop a high-performance PTE-predictive ANN model based on other studies and risk factors available in clinical practice.

### Advantages of the ANN Model

ANN models are able to model nonlinear relationships between input and output variables in a high-dimensional dataset and select the optimal model with high accuracy. ANN models have been widely used to predict the occurrence of hypertension [34] and mortality in patients with stroke [35]. ANN models excelled in many ways compared to conventional statistical methods; for example, they have higher classification accuracy and a better ability to analyze nonlinear relations and handle correlated independent variables [26].

Existing PTE prediction models are mainly risk scoring models built by traditional statistical methods. In our previous work, we set up a nomogram model to predict the risk of PTE. This model consisted of 7 risk factors (sex, TBI severity, duration of loss of consciousness time, subdural hematoma, early PTS, contusion site, and treatment) found in multivariable Cox proportional hazards regression analysis based on the same training cohort (West China Hospital). Our results showed that the AUC of this nomogram prediction model was 0.859 (95% CI 0.826-0.891), lower than the ANN model. In addition, with multivariable logistic regression and based on 9 significant risk factors (subdural hematoma, contusion load, craniotomy, craniectomy, seizure during acute hospitalization, duration of posttraumatic amnesia, preinjury mental health treatment/psychiatric hospitalization, intraparenchymal fragment, and preinjury incarceration), Ritter et al [36] constructed prognostic models to predict PTS during different times following TBI. Their results indicated that the corrected concordance statistics (equal to AUC) were 0.599, 0.747, and 0.716 for acute hospitalization, year 1, and year 2 models, respectively. In our study, we established an ANN model for PTE prediction using comprehensive data from training and 2 sets of tests that achieved AUC of 0.907, 0.867, and 0.859, respectively, higher than the existing models. In addition, Ritter et al [36] tested their model against subjects selected in bootstrap samples, while our ANN model was tested by the other 2 cohorts who were unaware of the training process. Our prediction model outperforms the abovementioned predictive models built by logistic regression method, which suggests that the ANN models have superiority and rationality in solving complex nonlinear relationships.

The new ANN model based on demographic and clinical data can be used as a simple screening tool to identify individuals at high risk of PTE after TBI. The predictors included in the model are common and available in routine practice. Beyond that, this model was tested by 2 cohorts and its performance was good, indicating that it might be applicable to the general population. In our study, we input 21 variables into the ANN model to predict the risk of PTE; all of these variables were mentioned in previous studies, while only some factors were considered as predictors of PTE by logistic regression. The ANN method has the advantage of feature selection over conventional statistical methods; when more factors are taken into account, the prediction is more accurate.

### Impact of the ANN Model in the Future

The ANN model has higher prediction accuracy and can contribute to future clinical decisions. It helps clinicians identify patients with high risk of PTE, so doctors follow them more closely after discharge and follow up more frequently for more precise personal management. Furthermore, the new model is also conducive to the selection of the target group for PTE prevention study. For example, by applying presumed data, a provider could estimate a TBI patient’s risk of PTE in the future. By studying the high-risk population predicted by the ANN model, it may be easier to find useful preventive measures. In addition, the ANN model can help clinicians conduct some trials on antiepileptic drug withdrawal. If according to the ANN model, the patient’s PTE risk is low and meets the withdrawal criteria, the clinician may try to withdraw the patient’s antiepileptic drug, which will reduce the financial burden and adverse effects of antiepileptic drugs.

### Limitations

However, there were some limitations to this study. First, we developed the ANN model using epidemiological data, mainly including demographic data, clinical manifestation, and radiological results, regardless of relevant laboratory data such as electroencephalogram. Second, this was a retrospective study that was prone to bias, and some of the factors in other studies have not been collected, such as whether a patient was mentally ill. Third, most of the factors included were dichotomous variables rather than continuous variables. The lack of dose-response relationship between exposure levels of these risk factors and PTE may not reveal their true relationships with PTE. Fourth, the ANN model relied more on computers and specific programs, so its application was not as convenient and simple as nomogram models for the clinicians [37].

Despite these shortcomings, as far as we know, this is the first study using ANN to predict the risk of PTE after TBI. Our results indicated that ANN analysis may be more accurate in predicting the incidence of PTE for individual patients than traditional statistical methods, and therefore the ANN model could help determine the use of antiepileptic drugs for individual TBI patients.

### Conclusions

In conclusion, our study was the first one to develop an ANN with a higher level of accurate prediction of PTE than the nomogram prediction model and other models constructed by multilogical regression. With the new ANN model, we can identify TBI patients at high risk of PTE as early as possible, and the model-predicted risk probability is significant for the selection of study population to determine the beneficial prevention and management of these PTE patients.

## Abbreviations

ANN: artificial neural network
AUC: area under the curve
ICD-10: International Statistical Classification of Diseases and Related Health Problems, Tenth Revision
NPV: negative predictive value
PPV: positive predictive value
PTE: posttraumatic epilepsy
PTS: posttraumatic seizure
ROC: receiver operating characteristic curve
TBI: traumatic brain injury
